# Circulating Betatrophin Correlates with Triglycerides and Postprandial Glucose among Different Glucose Tolerance Statuses—A Case-Control Study

**DOI:** 10.1371/journal.pone.0133640

**Published:** 2015-08-06

**Authors:** Ting Gao, Kairui Jin, Peihong Chen, Hua Jin, Lili Yang, Xinmiao Xie, Meili Yang, Cheng Hu, Xuemei Yu

**Affiliations:** 1 Department of Endocrinology and Metabolism, Fengxian Central Hospital, South District of Shanghai Jiao Tong University Affiliated Sixth People’s Hospital, Shanghai, China; 2 Department of Clinical Medicine, Fudan University Shanghai Medical College, Shanghai, China; 3 Shanghai Diabetes Institute, Shanghai Key Laboratory of Diabetes Mellitus, Shanghai Clinical Center for Diabetes, Shanghai Jiao Tong University Affiliated Sixth People's Hospital, Shanghai, China; University of Catanzaro Magna Graecia, ITALY

## Abstract

**Purpose:**

Previous researches of betatrophin on glucose and lipids metabolism under insulin-resistant condition have reached controversial conclusions. To further identify the possible impact of betatrophin, we measured the circulating betatrophin levels in newly diagnosed type 2 diabetes (T2DM) patients, and in subjects with both impaired glucose tolerance (IGT) and normal glucose tolerance (NGT) and investigated the relationship between serum betatrophin and other clinical parameters in these patients with different glucose tolerance statuses.

**Methods:**

A total of 460 permanent residents of the Fengxian District, aged 40–60 years, were enrolled. Based on the results of a 75 g oral glucose tolerance test, we selected newly diagnosed T2DM (n = 50) patients and subjects with IGT (n = 51) and NGT (n = 50) according to their age, gender and body mass index (18–28 kg/m^2^). Anthropometric parameters, glycosylated haemoglobin, blood lipids and fasting insulin were measured. Serum betatrophin concentrations were determined via ELISA.

**Results:**

Serum betatrophin levels in T2DM patients were increased significantly compared with IGT and NGT groups, and decreased in subjects with better islet beta cell function. Serum betatrophin was positively correlated with triglyceride, 2-hour postprandial glucose, alanine aminotransferase and aspartate transaminase after adjusting for age, sex and body mass index in all subjects. Multiple regression analysis showed that 2-hour postprandial glucose was independently associated with serum betatrophin significantly.

**Conclusions:**

Circulating betatrophin is increased in newly-diagnosed T2DM patients and positively correlated with the triglycerides and postprandial glucose levels. The results suggest that betatrophin may participate in glucose and triglycerides metabolism.

## Introduction

The prevalence of diabetes mellitus, which is a major public health issue, has been increasing dramatically over the last decade. In China, the prevalences of diabetes and prediabetes were 9.7% and 15.5% respectively, corresponding to 92.4 million adults with diabetes and 148.2 million adults with prediabetes according to a 2007 survey [1], and most of them were diagnosed with type 2 diabetes mellitus (T2DM). T2DM is accompanied by a cluster of serious complications including atherosclerosis, diabetic nephropathy and diabetic retinopathy, and leads to increased morbidity and mortality, resulting in a substantial burden and intangible cost to society. T2DM is characterized by beta cell dysfunction and insulin resistance. However, the available therapies forT2DM cannot prevent the gradual loss of beta cell function [2–3], and strategies to promote islet beta cell proliferation are therefore the focus of much attention.

Betatrophin, also known as lipasin or angiopoietin-like protein8 (ANGPTL8), is a 22kD hormone that is primarily expressed in the liver and adipose tissue. Recently, in a mouse model of insulin resistance developed via the administration of S961, Yi observed an increase in islet beta cell proliferation induced by the over-expression of betatrophin [4]. However, Gusarova [5] raised doubt by revealing that over-expression of angptl8 in mice doubles plasma triglyceride levels, but does not alter beta cell expansion. In clinical study, Espes observed an increased circulating betatrophin concentration in patients with long-standing type 1 diabetes [6], and studies on T2DM demonstrated that serum betatrophin was also increased in T2DM patients [7–8]. However, Gomez-Ambrosi [9] reported that serum betatrophin concentrations were decreased in patients with obesity and T2DM and Fenzl [10] reported that serum betatrophin presented no correlation with different glucose tolerance status but correlates with lipid profiles in patients with obesity or T2DM. Thus, no consistent conclusion has been reached thus far.

To further investigate the association of circulating betatrophin concentration with glucose and lipid metabolism, we measured betatrophin concentrations in subjects of newly-diagnosed T2DM, normal glucose tolerance (NGT) and impaired glucose tolerance (IGT) and analyzed the relationship between betatrophin and related clinical parameters. Our hypothesis was that serum betatrophin levels would increase in T2DM patients as compensation for the loss of beta cell function. An increase in serum betatrophin was observed in T2DM patients and serum betatrophin levels were correlated with both postprandial glucose and lipid levels.

## Materials and Methods

This study was approved by the Ethics Committee of Fengxian Central Hospital, and was carried out in accordance with the principles of the Declaration of Helsinki as revised in 2000. All subjects provided written informed consent.

### Subjects

A total of 460 permanent residents of the Fengxian District of Shanghai aged 40–60 years were enrolled (160 males, 300 females). Participants previously diagnosed with diabetes; special types of diabetes; acute and chronic inflammatory disease; heart, liver and lung disease; Cushing's syndrome and other diseases and on hypoglycaemic agents were excluded. Of the 460 individuals, 63 were newly diagnosed with T2DM, 108 with IGT and 289 with NGT based on oral glucose tolerance tests (OGTT) (WHO criteria, 1999). A total of 50 T2DM, 51 IGT and 50 NGT subjects were chosen to form matched groups in terms of age, gender and body mass index (BMI, 18–28 kg/m^2^).

### Anthropometric measurements

Weight and height were measured, and BMI (kg/m^2^) was calculated by dividing the weight (kilograms) by the squares of the height (meters squared). Waist circumferences were measured at the narrowest point between the lowest rib and the uppermost lateral border of the right iliac crest, the hips were measured at their widest point, and the waist-to-hip ratio (WHR) was calculated. Seated blood pressure was taken twice after the subjects rested for 10 min.

### Serum biochemistry

Blood samples were collected after an overnight fast in the morning and 2 hours after 75 g of glucose was taken. After clotting, the blood specimens were separated via centrifugation. The serum samples were subsequently stored in aliquots without preservatives at -80°C prior to the analysis of betatrophin. Fasting plasma glucose (FPG), 2-hour postprandial glucose (2hPG), triglycerides (TG), total cholesterol (TC), low-density lipoprotein (LDL-c), high-density lipoprotein (HDL-c), alanine aminotransferase (ALT) and aspartate transaminase (AST) were determined using an automatic biochemical analyser (Beckman DXC800, USA). Fasting insulin levels were determined through direct chemiluminescence (ADVIA Centaur automatic immunity analyser and the associated kit, Siemens), and glycosylated haemoglobin A1c (HbA1c) was determined via high performance liquid chromatography (HLC-723 G7 automated glycosylated haemoglobin analyser, Japan). Additionally, the homeostasis model assessment of insulin resistance (HOMA-IR) = (fasting insulin×fasting glucose)/22.5 and homeostasis model assessment-β(HOMA-β) = 20×fasting insulin/(fasting glucose-3.5) were employed. Body composition was analysed using a bioelectrical impedance analyser (INBODY 10, Korea).

### Betatrophin measurement

Fasting **s**erum betatrophin was determined using an ELISA kit (Wuhan Eiaab Science, Wuhan, China). A standard curve was drawn on logarithmic graph paper, with the concentration of the standard density on the horizontal axis (logarithmic scale) and the OD value on the vertical axis (logarithmic scale). Data analysis was carried out using the professional software Curve (Curve Expert 1.3), and standard curve regression equations were calculated based on the concentration of the standard density and the OD value. The concentrations of the samples were calculated using the sample OD value in the equation with multiplication by the dilution factor. All samples were analysed in duplicate.

### Statistical analysis

Statistical analysis was performed using SAS version 8.0. The data are presented as the mean ± SD or median (25^th^ and 75^th^ percentiles). The DBP, triglyceride, 2hPG, HOMA-IR, HOMA-β and betatrophin values were logarithmically transformed due to their non-normal distribution. Analysis of variance (ANOVA) was used to compare differences among the three groups. Student-Newman-Keuls (SNK) test was employed to compare differences between any two of the three groups. To further investigate the relationship between serum betatrophin and insulin resistance or beta cell function, we chose 75^th^ percentiles as cut points for HOMA-IR and HOMA-β, and divided the overall participants into two groups, respectively. Student *t* test was employed to compare the differences of betatrophin levels between the groups.

The correlations of betatrophin with the other variables were examined via Pearson’s bivariate correlation analysis. Although age, sex and BMI were controlled among the three groups, subjects of the three groups were not individually matched. To excluding the potential influence of these factors, multivariate regression was applied to adjust for age, sex and BMI as confounders and to investigate the independent influencing factors of betatrophin. Two-sided *P* values<0.05 were considered significant.

## Results

Anthropometric and biochemical characteristics of the individuals included in the study are shown in Table 1. Among the three groups, systolic blood pressure (SBP), triglycerides, FPG, 2hPG, HbA1c and HOMA-IR were significantly increased, whereas HOMA-β was significantly lower in the T2DM subjects (*P*<0.05 or *P*<1.0E-4). No significant differences in the waist, WHR, percentage of body fat (PBF), diastolic blood pressure (DBP), total cholesterol, HDL-c and LDL-c measurements were found among the three groups. Between NGT and IGT groups, 2hPG, HOMA-IR and HOMA-β were different significantly (*P*<0.05). Compared with NGT, SBP, TG, FPG, 2hPG, HbA1c and HOMA-IR significantly increased (*P*<0.05), and HOMA-β was significantly lower (*P*<0.05) in T2DM group. Compared with IGT, TG, FPG and HbA1c significantly increased (*P*<0.05) in T2DM group.

**Table 1:** Demographic and biochemical characteristics of the study population.

Variable	NGT(n = 50)	IGT(n = 51)	T2DM(n = 50)	*p* value
Age(years)	52.44±5.87	52.54±5.88	52.24±5.89	0.967
BMI(kg/m^2^)	24.48±2.00	24.39±1.91	24.70±2.14	0.733
Waist circumference(cm)	82.50±8.21	83.98±6.17	85.04±7.33	0.226
WHR	0.91±0.07	0.91±0.07	0.93±0.08	0.240
PBF(%)	32.28±5.74	32.73±4.34	32.54±5.06	0.908
DBP(mmHg)	80.00(70.00,86.00)	80.00(74.50,90.00)	80.00(76.00,90.00)	0.455
SBP(mmHg)	123.16±16.06†	127.79±15.61	131.78±16.44†	**0.032**
AST(IU/L)	24.66±1.465	21.80±0.941	26.30±1.851	0.834
ALT(IU/L)	27.72±2.576	25.82±1.606	40.72±6.375	0.523
TC(mmol/L)	5.23±1.11	5.16±1.03	5.47±1.13	0.337
TG(mmol/L)	1.16(0.77,1.70)†	1.31(1.01,1.96)‡	1.67(1.09,2.55)† ‡	**0.002**
HDL-c(mmol/L)	1.31±0.25	1.28±0.25	1.32±0.24	0.791
LDL-c(mmol/L)	3.06±0.82	2.95±0.72	3.16±0.85	0.423
FPG(mmol/L)	5.24±0.44†	5.76±0.59‡	7.93±2.71† ‡	**<1.0E-4**
2h-PG(mmol/L)	6.30(5.60,7.00)* †	8.50(8.10,10.10)* ‡	14.70(12.50,17.80)† ‡	**<1.0E-4**
HbA1c(%)	5.48±0.34†	5.85±0.44‡	7.11±1.89† ‡	**<1.0E-4**
HOMA-IR	1.58(1.19,2.55)† *	2.48(1.89,3.39)*	2.97(2.46,3.94)†	**<1.0E-4**
HOMA-β	85.10(61.10,131.57)† *	88.60(63.23,127.65) *	45.54(31.71,92.97)†	**<1.0E-4**

Data are shown as n or median (interquartile range). Statistical analyses were performed by SAS (version 8.0).*p* values from ANOVA, *p* values <0.05 are shown in bold.

*significantly different between IGT and NGT at P<0.05 from SNK.

^†^significantly different between T2DM and NGT at P<0.05 from SNK.

^‡^significantly different between T2DM and IGT at *P*<0.05 from SNK.

**Fig 1:**
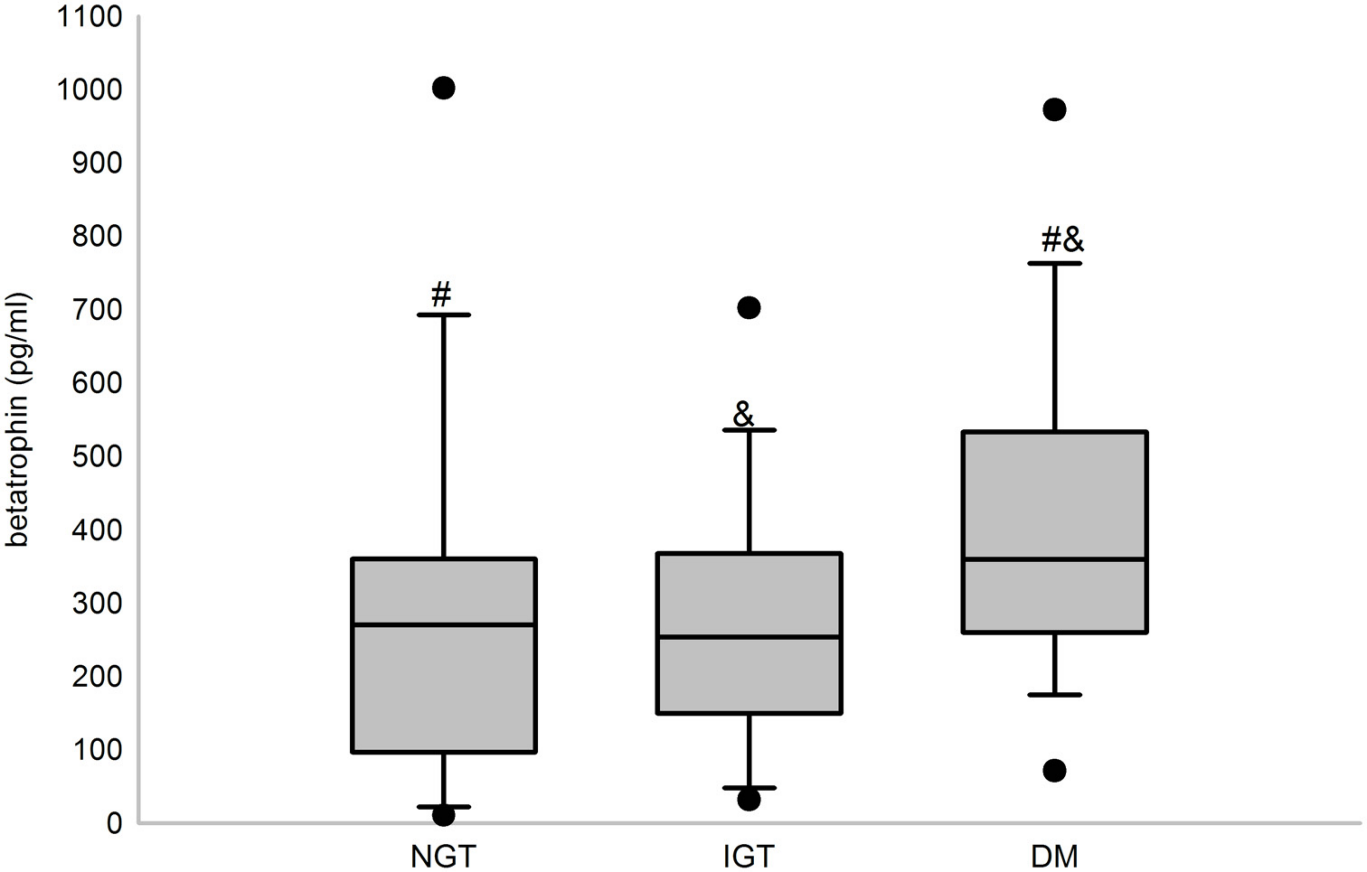
Serum betatrophin levels of subjects with different glucose tolerance statuses. Box plot for serum betatrophin levels in NGT (n = 50), IGT (n = 51) and T2DM (n = 50) subjects. Serum betatrophin levels were significantly different in the three groups (*P*<0.01). #significantly different between T2DM and NGT at *P*<0.05 from SNK; &significantly different between T2DM and IGT at *P*<0.05 from SNK.

**Fig 2:**
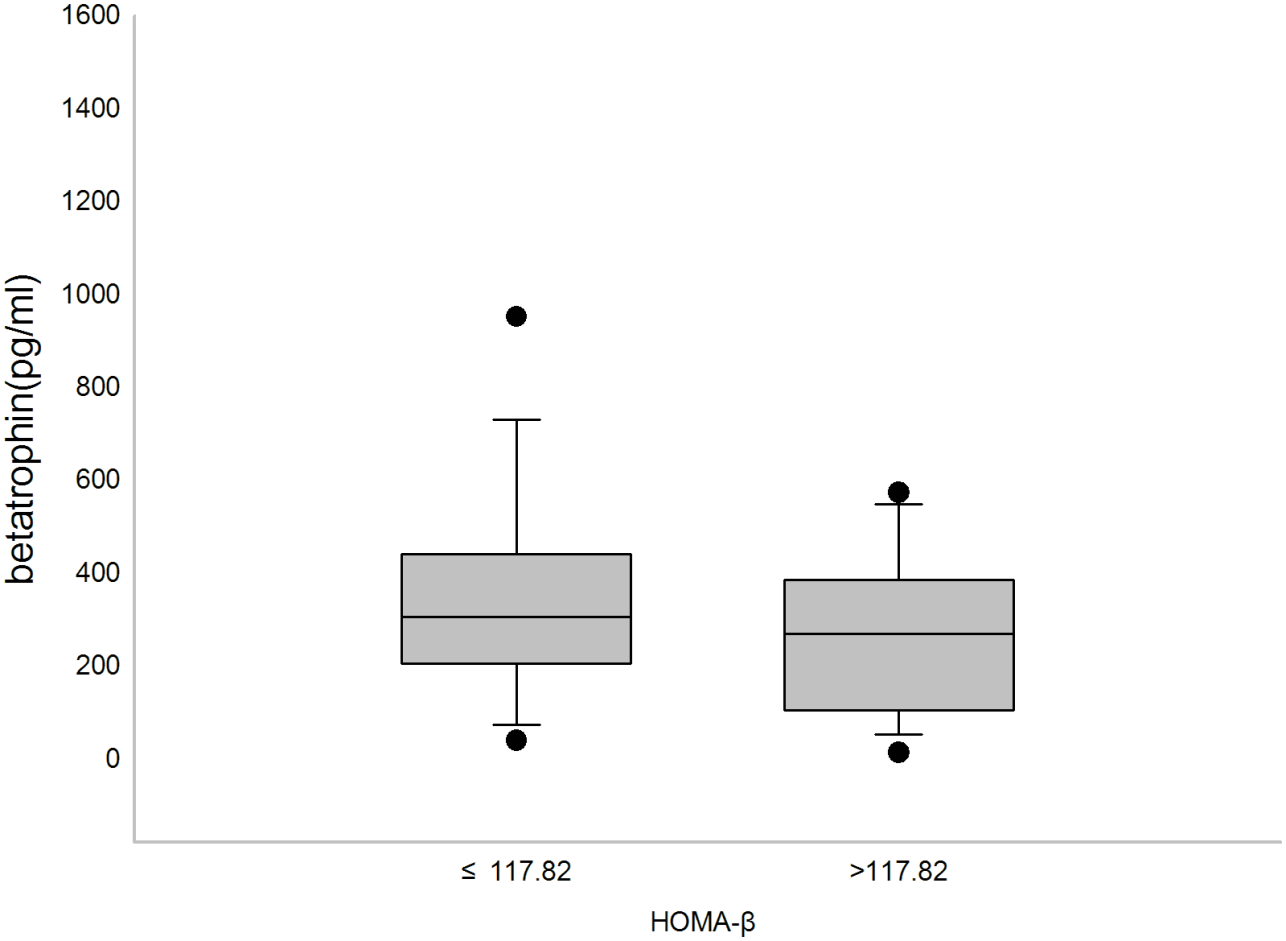
Serum betatrophin levels of subjects with different levels of beta cell function. Box plot for serum betatrophin levels in subjects with HOMA-β below or equal to 117.82 (Q3) and subjects with HOMA-β above 117.82. Serum betatrophin levels were significantly different between the two groups (*P*<0.05).

Serum betatrophin concentrations were significantly different among NGT, IGT and T2DM subjects (*P* = 0.003), with higher concentrations observed in the T2DM group than the NGT or IGT group (Fig 1) (*P*<0.05). However, no difference was detected between the NGT and IGT groups. In addition, serum betatrophin levels were significantly lower in subjects with comparatively better islet beta cell function (Fig 2) (*P*<0.05). However, no difference of betatrophin levels was found between subjects with different insulin resistant levels.

**Fig 3:**
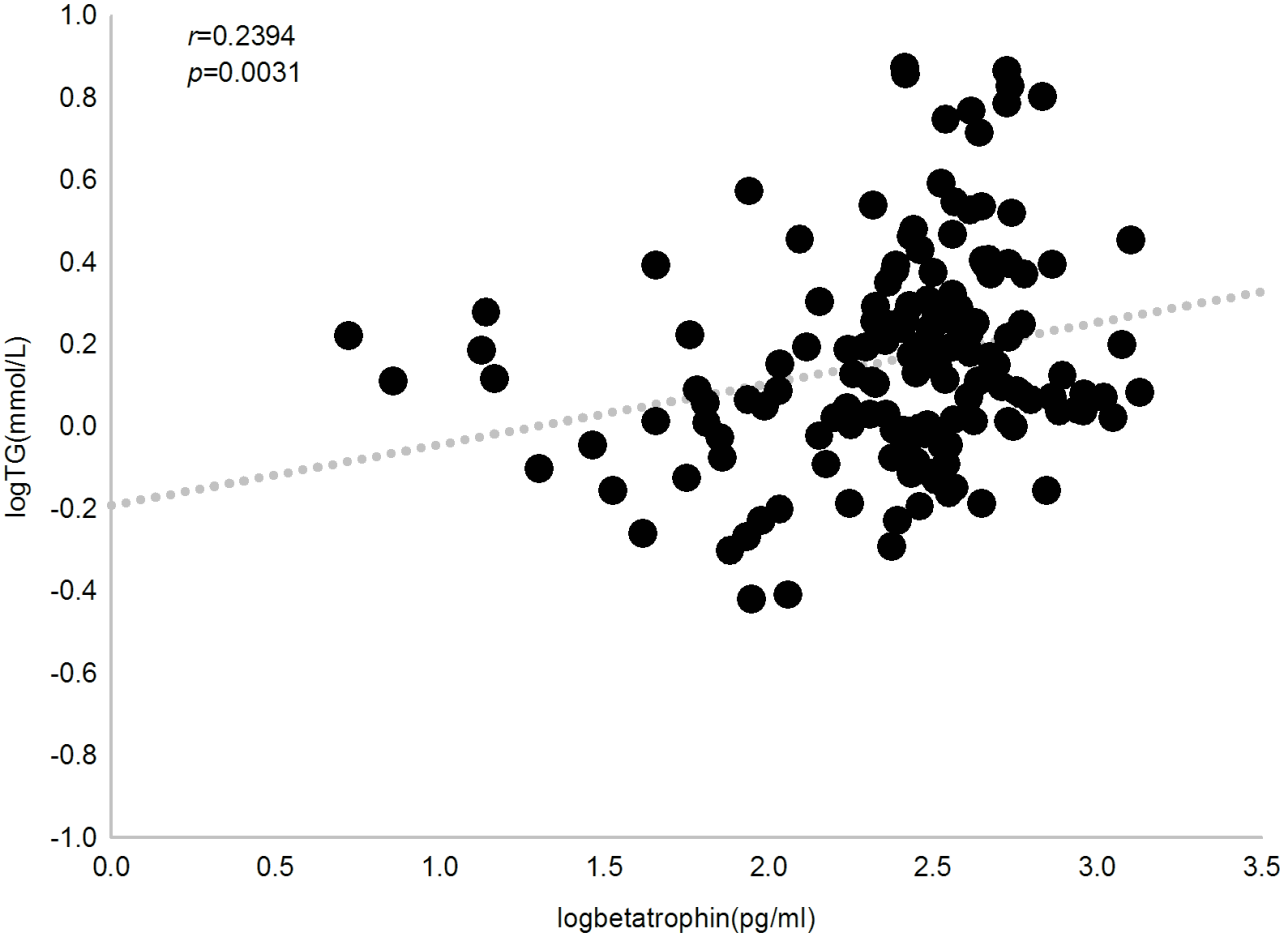
Correlation between plasma betatrophin concentration and triglyceride level. Betatrophin level positively correlates with triglyceride level in all subjects (*r* = 0.239, *P* = 0.0031).

**Fig 4:**
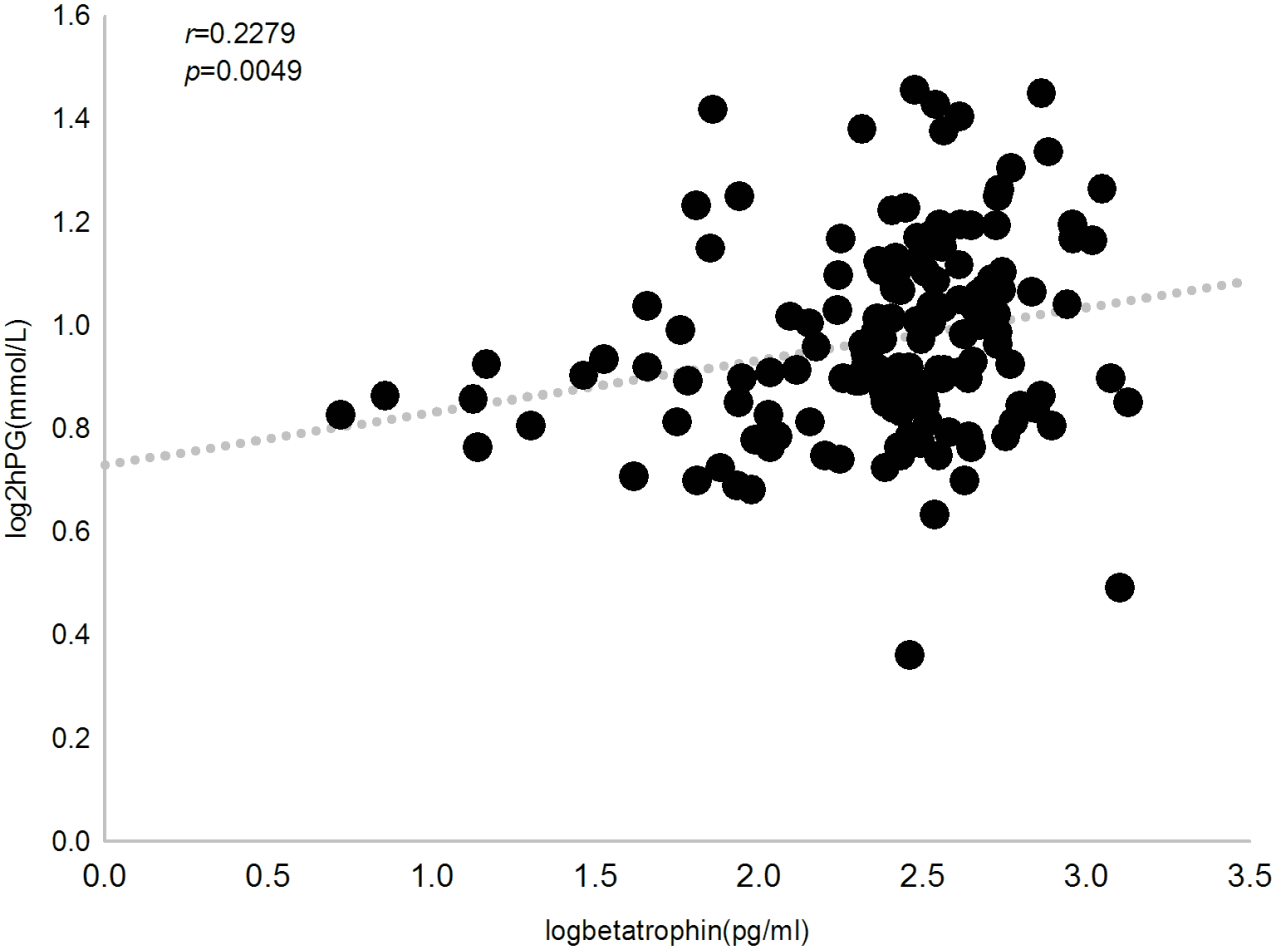
Correlation between plasma betatrophin concentration and 2-hour plasma glucose level. Betatrophin level positively correlates with 2-hour plasma glucose level in all subjects (*r* = 0.228, *P* = 0.0049).

**Fig 5:**
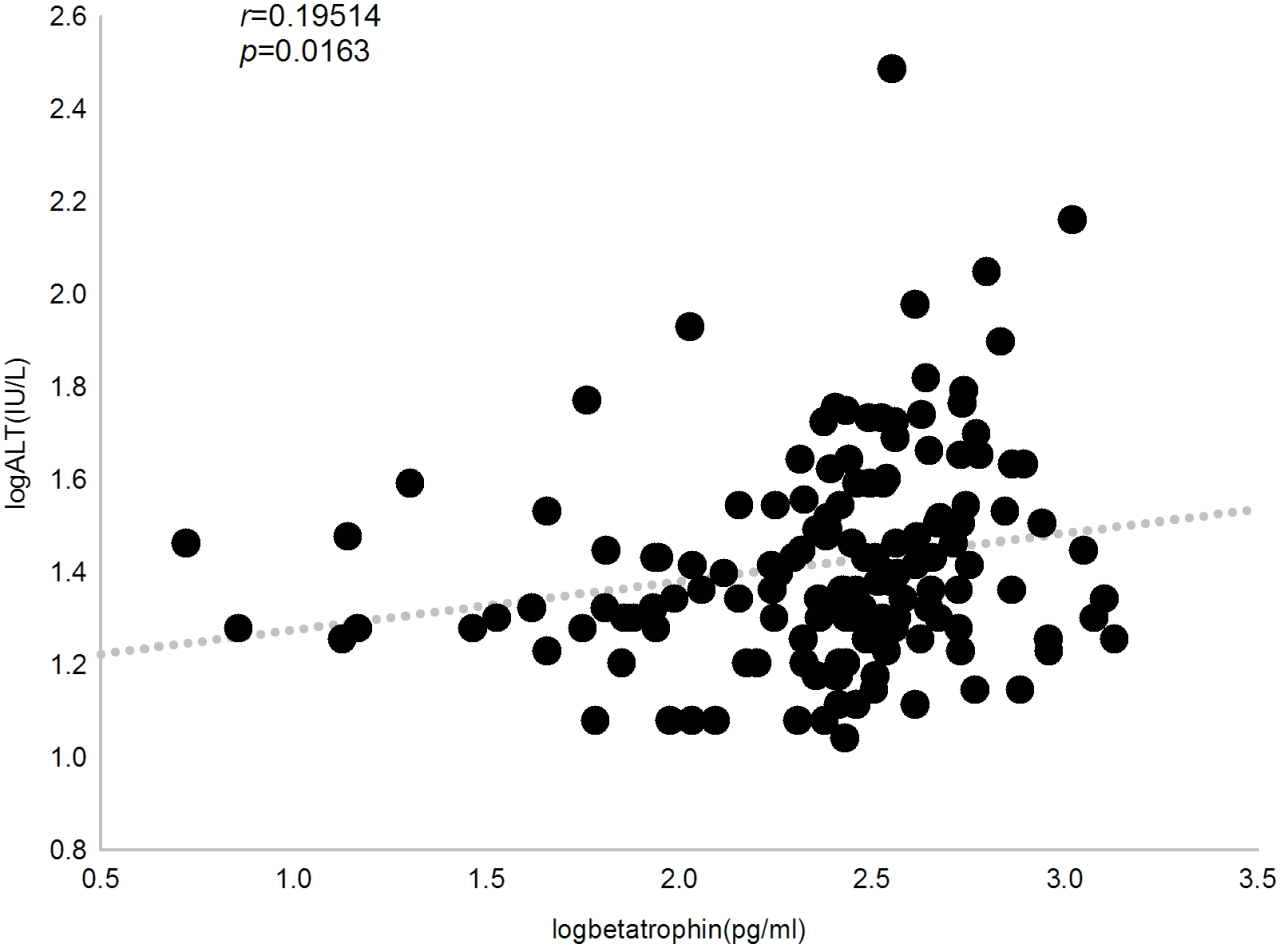
Correlation between plasma betatrophin concentration and alanine aminotransferase level. Betatrophin level positively correlates with alanine aminotransferase level in all subjects (*r* = 0.195, *P* = 0.016).

**Fig 6:**
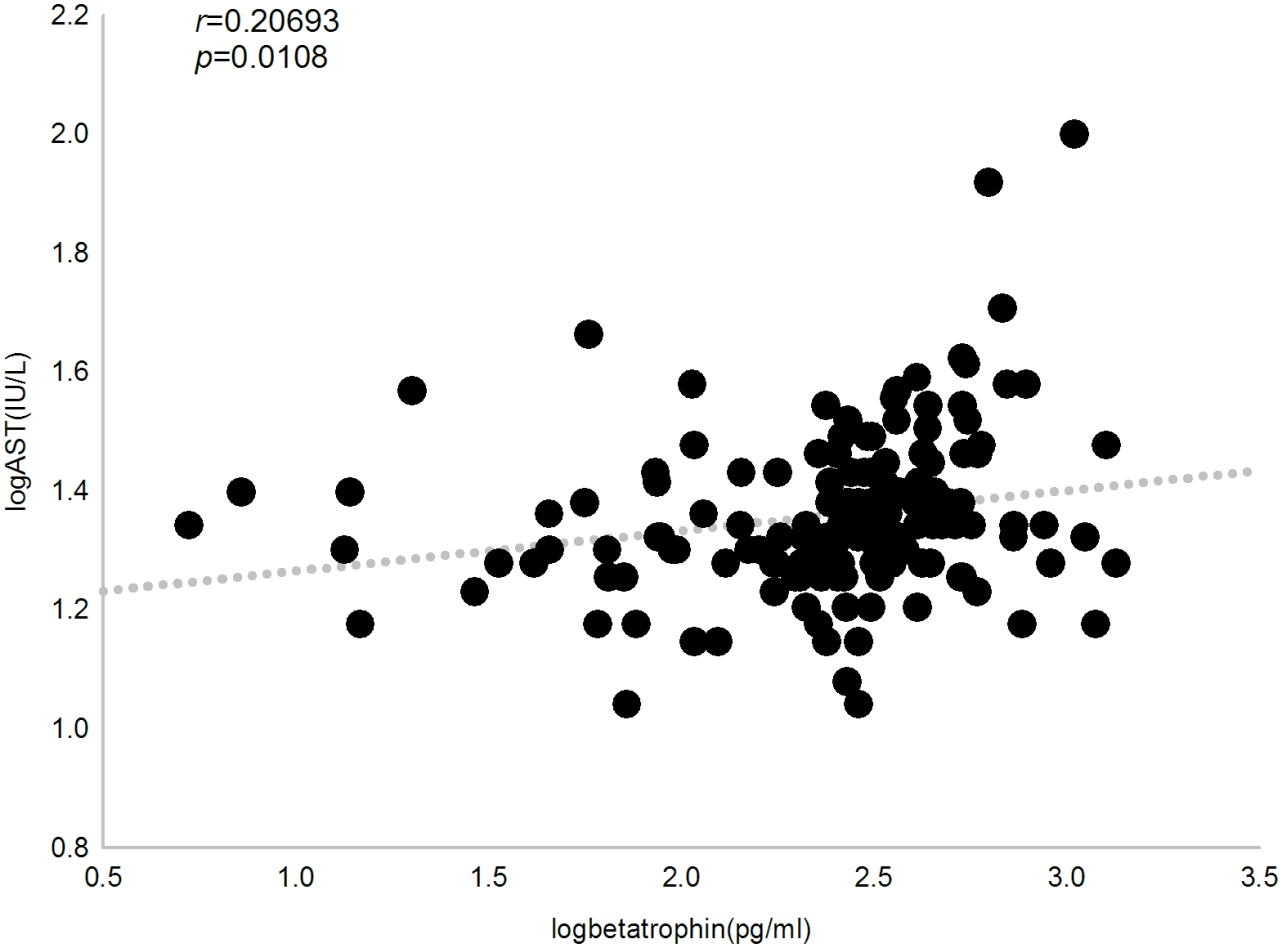
Correlation between plasma betatrophin concentration and aspartate transaminase level. Betatrophinlevel positively correlates with aspartate transaminaselevel in all subjects (*r* = 0.207, *P* = 0.011).

We then investigated the relationship between circulating betatrophin levels and clinical parameters by Pearson’s bivariate correlation analysis. In all subjects, betatrophin concentration was positively correlated with triglyceride, 2hPG, ALT, AST and ALP (*r* = 0.239, 0.228, 0.195, 0.207 and 0.183, respectively, *P*<0.05). When multivariate regression was applied and adjusted for age, sex and BMI as confounders, we observed that serum betatrophin levels were independently and significantly associated with triglyceride (*P* = 0.012), 2hPG (*P* = 0.004), ALT (*P* = 0.0388) and AST (*P* = 0.0306) (Figs 3, 4, 5 and 6).

Finally, we used multiple regression analysis to investigate the independent factors associated with betatrophin. The results showed that 2hPG (β = 0.386±0.189, *P* = 0.0425) was the only independent factor that was significantly associated with serum betatrophin.

## Discussion

We analysed the serum levels of betatrophin in middle-aged, newly diagnosed T2DM patients and compared them with age-, gender- and BMI-matched IGT and NGT subjects. The results showed that serum betatrophin concentrations were increased in newly diagnosed T2DM patients, but no difference was observed between the NGT and IGT groups. In addition, serum betatrophin levels were decreased in subjects with better islet beta function. Circulating betatrophin was positively correlated with triglycerides, 2hPG, FPG and HbA1c. Betatrophin was independently and significantly associated with triglyceride and 2hPG after adjusting for age, sex and BMI. These results indicate that betatrophin may participate in triglyceride and glucose metabolism. We also observed that betatrophin positively correlated with particular liver enzymes-ALT and AST specifically.

Our results demonstrated a significant increase in serum betatrophin levels of newly diagnosed T2DM patients compared with the controls, which accorded with several previous studies [7–8, 11–12]. However, Gomez-Ambrosi and Fenzl recently reported that serum betatrophin showed no increase in T2DM patients [9–10]. This disparity may be due to sample size, hypoglycaemic agents and disease duration and disparity of BMI. In the study by Fenzl, all of the patients with T2DM received metformin and/or sulfonylurea and the mean disease duration was 8.3±1.0 years. In the study of Fu, the BMI of the subjects varied from 23.6±1.2 to 39.0±3.6. Whether these factors affect serum betatrophin levels is still not clear.

Our results also showed that 2hPG was the independent factor influencing betatrophin which indicates a probable impact of betatrophin in the pathogenesis of T2DM, but the mechanism remains exclusive. Yi reported that under the condition of insulin resistance induced by S961 (an insulin receptor antagonist), hepatic betatrophin mRNA expression was upregulated and correlated with high pancreatic beta cell proliferation rates. Betatrophin encoded a secreted protein which was proved to be responsible for the proliferation of pancreatic beta cell in animal models [4]. Nevertheless, the potential receptors and molecular mechanism of betatrophin still remain uncovered. This increase might be an indication of the ability to adapt to insulin resistance and a compensation for beta cell loss. In addition, the increase of serum betatrophin in subjects with poorer beta cell function could be seen as a compensation of beta cell loss.

However, controversy emerges regarding the role of betatrophin in glucose metabolism. Jiao showed that elevated hepatic betatrophin expression promoted the replication of mouse beta cells but not human beta cells transplanted into mice [13]. Gusarova reported that angptl8 knockout mice underwent entirely normal beta cell expansion in response to insulin resistance, and over-expression of ANGPTL8 either did not alter or only modestly increased beta cell replication, indicating that there may be unknown factors responsible for the variation of beta cell replication in over-expression of betatrophin [5]. Yi and colleagues responded to the findings of Gusarova and reported that they also found great variation in beta cell replication following the over-expression of betatrophin in mice [14]. Recent study of the relationship between betatrophin and irisin indicates that there might be other factors participating in this process [15] and study on the molecular mechanism of beta cell proliferation suggests that there might be a pathway involving both irisin and betatrophin in insulin resistance [16]. Therefore, studies on betatrophin are still in the nascent stages.

In the present study, serum betatrophin levels showed a positive correlation with triglycerides, illustrating that betatrophin might plays a role in regulating lipid metabolism. In fact, previous studies have shown that betatrophin regulates lipid metabolism. Ren found that the RIFL (refeeding induced in fat and liver) gene (a betatrophin gene) was important in fat metabolism and was specifically expressed in white adipose tissue and the liver following refeeding in fasting mice [17]. ANGPTLs play major roles in lipids metabolism, and ANGPTL8 regulates postprandial triglycerides and fatty acid metabolism in mice [18]. Angptl8 (-/-) knockout mice gained weight slowly, and their plasma triglycerides decreased after refeeding [19]. The proposed mechanism of these phenomena is that betatrophin inhibits lipoprotein lipase (LPL), thereby inhibiting triglycerides being taken up by peripheral tissues and reducing serum triglyceride clearance [20–21]. As a result, over-expression of betatrophin leads to hypertriglyceridemia. Thus, betatrophin is a nutritionally regulated factor that plays a role in lipid metabolism.

We also observed a positive correlation between betatrophin and liver enzymes, ALT and AST in particular, which coordinate with the results of Chen [12]. However, the reason for that is merely known. As discussed previously, serum betatrophin level positively correlated with serum triglycerides, and it is widely acknowledged that hypertriglyceridemia is closely related to non- alcohol fatty liver disease which can cause an increase in liver enzymes. Therefore, betatrophin may have an impact on the pathogenesis of non- alcohol fatty liver by increasing serum triglyceride level.

A main limitation of the present study is the limited sample size, which reduces the statistical power. Second, postprandial levels of betatrophin could not be analysed because only fasting betatrophin was measured. Third, serum betatrophin levels were determined only via ELISA, without verification by western blotting.

We conclude that circulating betatrophin levels are increased in newly diagnosed T2DM patients and decreased in patients with comparatively better islet beta cell function. Serum betatrophin levels show a positive correlation with triglycerides and 2hPG, indicating that betatrophin is associated with both glucose and lipid metabolism. However, we cannot reach conclusions on the role of betatrophin in glucose and lipid metabolism process based on the present results and further studies will still be needed.
